# The Effects of Working Memory Capacity in Metaphor and Metonymy Comprehension in Mandarin–English Bilinguals’ Minds: An fMRI Study

**DOI:** 10.3390/brainsci12050633

**Published:** 2022-05-11

**Authors:** Chia-Hsin Yin, Fan-Pei Gloria Yang

**Affiliations:** 1 Department of Teaching and Learning, The Ohio State University, Columbus, OH 43210, USA; yin.762@osu.edu; 2 Department of Foreign Languages and Literature, National Tsing Hua University, Hsinchu 300044, Taiwan; 3 Center for Cognition and Mind Sciences, National Tsing Hua University, Hsinchu 300044, Taiwan; 4 Department of Radiology, Graduate School of Dentistry, Osaka University, Osaka 565-0871, Japan

**Keywords:** metaphor, metonymy, working memory, vocabulary, functional magnetic resonance imaging (fMRI)

## Abstract

This study investigated the role of working memory capacity (WMC) in metaphoric and metonymic processing in Mandarin–English bilinguals’ minds. It also explored the neural correlations between metaphor and metonymy computations. We adopted an event-related functional magnetic resonance imaging (fMRI) design, which consisted of 21 English dialogic sets of stimuli and 5 conditions: systematic literal, circumstantial literal, metaphor, systematic metonymy, and circumstantial metonymy, all contextualized in daily conversations. Similar fronto-temporal networks were found for the figurative language processing patterns: the superior temporal gyrus (STG) for metaphorical comprehension, and the inferior parietal junction (IPJ) for metonymic processing. Consistent brain regions have been identified in previous studies in the homologue right hemisphere of better WMC bilinguals. The degree to which bilateral strategies that bilinguals with better WMC or larger vocabulary size resort to is differently modulated by subtypes of metonymies. In particular, when processing circumstantial metonymy, the cuneus (where putamen is contained) is activated as higher-span bilinguals filter out irrelevant information, resorting to inhibitory control use. Cingulate gyrus activation has also been revealed in better WMC bilinguals, reflecting their mental flexibility to adopt the subjective perspective of critical figurative items with self-control. It is hoped that this research provides a better understanding of Mandarin–English bilinguals’ English metaphoric and metonymic processing in Taiwan.

## 1. Introduction

### 1.1. Research Background

#### 1.1.1. Figurative Language

Figurative language, e.g., idioms (e.g., beat around the bush), metaphors (e.g., knowledge is power), and metonymies (e.g., the pen is mightier than the sword), combines semantics and world knowledge with abstract reasoning and is used to convey thoughts, feelings, and ideas that may be inexpressible or less effectively expressed with literal language [1]. Therefore, figurative language competence indicates the ability of abstract thinking [2] and higher-level language processing. In particular, metaphor and metonymy are familiar forms of figurative language and crucial in our everyday language [3,4,5]. Such nonliteral expressions are widely used to express symbolism in the arts [6,7].

The issue of hemispherical processing for figurative language comprehension is still a debate. Research on the neural correlates of metaphor comprehension has historically focused on the role of right hemisphere (RH) contributions. Early neuropsychological research has suggested that the RH is necessary for understanding figurative speech [8,9], which is supported by some previous neuroimaging research [10,11]. However, another set of neuroimaging studies have proposed the increasing importance of left hemisphere (LH) involvement in metaphor comprehension [12,13,14,15]). A meta-analysis of neuroimaging investigations of discourse even found that there was no evidence of RH contributions to metaphor processing that were unaccompanied by LH contributions [16]. On top of that, Benedek et al. was the first to investigate the neural correlations of figurative language production, claiming that the generation of novel metaphors particularly relies on the left angular gyrus (AG) and the posterior cingulate cortex (PCC), and the left dorsomedial prefrontal (DMPFC) is activated by both metaphor production and creation [17]. Lai and Desai further proposed that there is no lateralization effect for metaphors; both hemispheres are statistically equally activated with small numerical right lateralization [18]. In line with the “spill over” effect in the RH, the LH is activated more for all types of difficult linguistic stimuli. Lai and Desai suggested that the right inferior frontal gyrus (IFG), especially, is activated when the resources provided by the LH are not sufficient due to comprehension difficulties [18].

On the other hand, the neural basis for metonymic processing seems to be a new topic. Few previous attempts have discussed this issue. Recent behavioral research [19] with self-paced reading (SPR), event-related potentials (ERP), and functional magnetic resonance imaging (fMRI) has begun to shed light on the cognitive abilities underlying metonymy, such as referential dependency. Piñango and colleagues proposed that two types of metonymies (systematic and circumstantial) share an underlying processing mechanism with overlapping activation in the cortical regions, especially the ventrolateral prefrontal cortex (vlPFC) and the dorsolateral prefrontal cortex (dlPFC) [19]. This mechanism differs in the contextualization needed. More recently, in a study that looked at German idioms, Michl [20] further pointed out that metonymies are perceived as less non-literal metaphorical idioms and that metaphors are generally highly non-literal. Besides, metonymy is based on more basic cognition and is suggested to be easier to acquire and comprehend than metaphorical language [21,22] in both children [23] and seniors [24,25,26]. This idea is also supported linguistically. Metonymies function within one semantic concept or domain, as opposed to metaphors which connect distinctive semantic concepts and domains [27,28]. Namely, “what is said is likely cognitively or semantically closer to what is meant in a metonymy than in a metaphor (Michl, 2019, p. 100, [20])”, which seems to suggest a systematic difference between metonymies and metaphors.

The overlapping process between WM and figurative language competence [29,30] has mainly been demonstrated in adults [31]. More specifically, WM mediates the ability to integrate and infer meaning in understanding figurative language, since non-literal processing is supposed to utilize contextual information to construct meaning [2,32] as well as to defer a literal interpretation [2]. In a study of WM involvement with ambiguity resolution [33,34], a three-stage model comprising the metaphor comprehension of determination, checking, and the reinterpretation of the literal meaning to derive a conveyed meaning was posited to allow for nonliteral comprehension [35]. Qualls and Harris [30] showed that adults’ WM and reading comprehension skills should be considered in metaphor comprehension, while coherent interpretation is more likely to appear when metaphors are contextualized and presented within contexts [36]. Similar to metaphor processing, metonymical comprehension is also context-bound [30], and it requires an increased memory load, inferential ability, and contextual cues to relevant information through determining the similarities between the metonymical targets [37]. Compared to the robust relationship between WM and metaphors, the study on the association between WM and metonymy is scarce.

Two further novel studies have explained how metaphor and metonymy are different, both cognitively and semantically. Fregni et al. [38] compared the neural mechanisms of metaphor and metonymy processing, albeit with the hearers’ understanding, demonstrating left-lateralized frontotemporal activation related to the theory-of-mind regions [39], such as the superior temporal gyrus (STS), the temporoparietal junction (TPJ), the medial prefrontal cortex (mPFC), and the precuneus, in both types of non-linear language. The study highlighted the possibility for a greater engagement of cognitive control and conflict resolution in both metaphor and metonymy comprehension. Nonetheless, metaphor resolution seems to have more flexibility for greater distance between the semantic source and the target domain. In terms of metonymy processing, the right inferior frontal was found, due to extra cognitive effort, to integrate nonliteral reference into the major meaning of the metaphorical sentence, which was consistent with Rapp et al.’s study [40]. However, these two rigorous research studies fail to fully address the bigger body of literature pertinent to metonymies, or the relationship between metaphorical and metonymic processing. There is a critical need to study the neural correlation between metaphor and metonymy in further detail.

Given that both metaphoric and metonymic comprehension require inferential processing [37,41,42], it is reasonable to assume that cognitive processing resource (i.e., working memory) demands will vary depending on the type of figurative language. In addition, researchers argue that WM is central to language comprehension since it is necessary for the integration of information and the resolution of ambiguity [33,34]. Although some progress has been made in understanding how metaphors are comprehended [43,44,45], the study of how metaphor is neuronally related to metonymy, working memory—an especially significant memory area for learning [46,47]—and vocabulary size, particularly in Chinese–English bilinguals, remains generally outside the mainstream of neurolinguistic investigation.

#### 1.1.2. Working Memory (WM)

Briefly, working memory (WM) is defined as the aspect of memory that involves the simultaneous storage and processing of information. Theoretically, WM is composed of three subcomponents: an executive attentional controller, a memory buffer for processing phonological information, and a memory buffer for processing visuospatial information [48,49]. The resource hypothesis proposes that WM comprises a limited number of general-purpose resources that can enable or enhance a range of cognitive functions [34], including reasoning, learning, mental calculation, and language comprehension [46,50]. Furthermore, WM is a significant component of the cognitive processes underlying bilingual language processing and the performance of second language proficiency [51]. Based on the Bilingual Anterior to Posterior and Subcortical Shift (BAPSS) model [52], due to the augmented demands on working memory and several language/executive control processes, more frontal network function should be established when an additional (L2) language is practiced. Shifts to the subcortical and posterior regions occur with more continuous and sustained exposure to an L2 in a bilingual immersion environment [53]. In other words, WM is critical to bilingualism.

Neuroimaging research has demonstrated that the neurobiological basis of the WM procedure is known to involve the prefrontal cortex (PFC) [54,55,56]. For both verbal and visual working memory tasks, the prefrontal cortex and secondary visual cortex are activated bilaterally. The inferior frontal cortex, inferior parietal cortex, and temporal gyrus are principally activated in the left hemisphere [57], whereas predominantly right activation occurs in the inferior parietal region. The importance of the superior occipital gyrus in the visual short-term memory, during the verbal memory updating task, has also been identified [56]. In a functional MRI study [55], a bilateral activation in the dorsolateral prefrontal cortex (BA 46 and BA 9), as well as in the anterior cingulate during the concurrent performance of span tasks, which is expected to engage the central executive, was observed. In addition, Lewis et al. [58] and Monetta et al. [59] even suggest that Parkinson disease participants with impaired WM are simultaneously impaired in the processing of metaphorical language depending on their fronto-striatal systems for working memory.

#### 1.1.3. WM, Vocabulary, and Bilingualism

Notably, WM is also reflected in the inhibitory control areas associated with bilingualism and is studied in relation to vocabulary size [60,61,62]. In a Chinese–English bilingual fMRI study, Guo, Liu, Misra, and Kroll [63] concluded the recruitment of different systems for each local inhibition, including the dorsal anterior cingulate cortex (ACC) and the supplementary motor area (SMA). They demonstrated that the dorsal left frontal gyrus and parietal cortex play a role in global inhibition. Since bilinguals must constantly regulate attention [64] between two simultaneously active language systems [65,66,67], the attentional control and executive functioning related to WMC is more developed in bilingual children than in their monolingual counterparts in terms of selective focus on target hints in conflicting situations [64]. Intriguingly, according to Bialystok et al. [60] and Bialystok and Luk [61], the effects of bilingualism on vocabulary size show consistency in age, yet this claim was only based on their recruitment of native English speakers for the study. In the same investigation of the vocabulary size of bilingual children [60] and adults [61], Bialystok and colleagues [60] reported that bilingual children possess a smaller lexicon compared to monolingual speakers. In a similar vein, using the Peabody Picture Vocabulary Test [68], adults were found to control a smaller vocabulary community than their monolingual equivalents [61,69]. Prat et al. [70] stressed that individuals with a smaller lexicon and lower WMC resort to “neural efficiency” strategy to draw more RH neural resources for metaphor comprehension, which indicates that more attentional demands or conflict monitoring processing would be needed for increased anterior cingulate activation. This research corroborates that vocabulary size is more strongly related to neural efficiency than individual differences in WMC to neural efficiency [70,71].

The above arguments thus increased our curiosity about the potential effects of individual differences in vocabulary size, and how bilinguals process metaphors and metonymy. Another motive we have is that vocabulary knowledge and comprehension skills are correlated with verbal working memory and could be quality predictors for metaphor interpretations [72]. Receptive vocabulary ability has been proved to be a highly reliable predictor of metaphor and metonymy performance in children and adults [73]. Additionally, strengthening language learners’ metaphor awareness facilitates their vocabulary acquisition and retention [74]. Reading and vocabulary build the mechanism that constructs rich and complex semantic networks [75,76]. The richer our semantic networks are, the higher quality metaphors we produce. Furthermore, the higher scores in working memory measures also link to better inhibitory control and well-built metaphors [77]. On top of that, in this multilingual world, bilingualism and multilingualism are global trends [78] which have attracted abundant attention in the language acquisition discipline [79,80,81]. However, in the novel and regular metonymy tests [82], as expected, speakers of English, Korean, and Spanish treated the tasks differently, and the most proficient learners of English demonstrated the closest performance to native speakers. This result can be explained by the generative lexicon approach to metonymy and calls for more attention on non-English native speakers. The inquiry of whether bilingualism in relation to other languages, such as Mandarin Chinese, sheds lights on WMC and lexicon in understanding figurative language is underexplored.

#### 1.1.4. Types of Metonymies

Our interest in different types of metonymies has been inspired by the only neuro-cognitive study (to the best of our knowledge) in systematic and circumstantial metonymy [19] so far. Pinango et al. [19] illustrated the differences between the two labels of metonymies based on the different terminologies used to describe these labels: “whereas systematic metonymy (e.g., producer-or-product, place-for-event, place-for-inhabitant) has been given labels like regular polysemy or lexical metonymy [83,84], circumstantial metonymy has been given labels such as reference or meaning transfer [85,86])” (p. 352). In this study, an example to unveil the differences between the two types of metonymies is provided: (a) highly conventionalized systematic metonymy (producer-for-product: “We all read Shakespeare”); (b) less conventionalized circumstantial metonymy (“[an old bartender says to the new hire:] ‘The martini at table 10 is a regular customer.’”). Systematic metonymy requires more contextual demands, whereas circumstantial metonymy is more contextualized [19]. Both types capture a similar dependency between the named and intended conceptual aspects [19,85,86,87].

### 1.2. Research Purpose

As previously described, substantial psycholinguistic [75,88,89] and neuroscientific [10,13] research has thoroughly investigated the cognitive processes in metaphor comprehension. Nonetheless, little is known about how metaphor is related to metonymy, a conceptual projection whereby one domain is partially understood in terms of another domain included in the same experiential area [90]. As a result, the present study aims to identify the plausible relationship between metaphorical and metonymic sentence processing on a neural basis.

Additionally, individual differences in cognitive capacities are associated with differences in the recruitment and modulation of working memory and executive function regions [33,75], indicating the “overlapping computations in metaphor comprehension and general thinking and reasoning (p. 282, [70])”. Since metonymic comprehension also requires abstract thinking [2] and reasoning, the second purpose of this study is to investigate the relationship between individual differences in working memory capacity and metaphor and metonymy processing.

Alternatively, we could expect a common neural signature for all types of figurative language processing because they all require a certain amount of non-literal understanding that goes beyond literal expression. As predicted by the standard pragmatic model, all types of figurative language violate the maxim of truthfulness [91,92]. Potential regions for brain areas shared by all types of figurative language would, consequently, include regions that are associated with theory-of-mind processing [93]. However, comparison differences in different types of metaphors [94], such as between metaphor and irony [95] (Eviatar and Just [96]) or between metaphor and sarcasm [97], have been found predominantly in first-language English (L1) speakers. Accordingly, this study fulfils this gap and aims to further investigate whether the importance of working memory capacity [77] and vocabulary size [70] have an impact on either metonymy (systematic metonymy and circumstantial metonymy) [19] or metaphor computation, which, to our belief, has not been thoroughly investigated in Mandarin–English bilinguals. We therefore hypothesize that:

1. Metaphorical processing engages some crucial brain areas with metonymic processing;
2. There are individual differences in WMC for bilinguals when comprehending metaphor and systematic and circumstantial metonymy;
3. There are individual differences in vocabulary size for bilinguals when comprehending metaphor and systematic and circumstantial metonymy.

As such, we propose three research questions to guide the study:

1. What are the neural correlates between metaphor and metonymy processing in Mandarin–English bilinguals’ minds?
2. What is the role of working memory capacity in metaphor and metonymy in Mandarin–English bilinguals’ minds?
3. What is the role of vocabulary size in metaphor and metonymy comprehension in Mandarin–English bilinguals’ minds?

## 2. Materials and Methods

### 2.1. Participants

Data were initially collected from twenty-one Mandarin–English bilinguals (six males, fifteen females; mean age = 27.19; SD = 4.23) recruited through various universities in northern Taiwan. Of these, 4 participants were excluded because of excessive head motion (greater than 2 mm) during fMRI data analysis; the remaining 17 participants, comprising 5 males and 12 females (mean age = 27.11; SD = 4.56), were included for the whole-brain imaging analysis. All participants were right-handed and self-reported to have no history of neurological illness, brain injury, or psychiatric disease. They had normal or corrected-to-normal vision. Written informed consent, in accordance with the guidelines set by the National Tsing Hua University Research Ethics Committee and Imaging Center for Integrated Body, Mind, and Culture Research, National Taiwan University was obtained, and compensation fees were given to all participants.

### 2.2. Experiment Tasks

In addition to the scanner, a Language Background Questionnaire with selected parts from the Language Experience and Proficiency Questionnaire (the LEAP-Q) [98], a reading span task in Mandarin, a reading span task in English [99], and the Nelson–Denny Reading Test [100] were taken by the subjects.

Inside the scanner, the subjects undertook a valence judgement task under three intermixed conditions (literal, metaphor, and metonymy) of five language subtypes: systematic literal, circumstantial literal, systematic metonymy, circumstantial metonymy, and metaphor.

#### 2.2.1. The Language Background Questionnaire and The Language Experience and Proficiency Questionnaire (LEAP-Q)

The Language Background Questionnaire was designed anew; the LEAP-Q was adapted and utilized in this study in order to scrutinize subjects’ English language learning experience, background, and proficiency. The LEAP-Q was constructed to assess foreign language learning experience and proficiency profiles in second and other foreign languages, irrespective of the specific languages involved. The internal validity of the LEAP-Q, the criterion-based validity of the LEAP-Q, and the predictive relationships between self-reported measures and performance on standardized language tests, as well as predictive relationships between language history and self-reported proficiency levels, have been recognized [98]. However, in terms of English proficiency level, given that Taiwan is an English as foreign language (EFL) context, which differs from an English as a second language (ESL) environment described in the LEAP-Q, we created the Language Background Questionnaire. Participants who self-reported reaching the CEFR—The Common European Framework of Reference for Languages: Learning, Teaching, Assessment, B2—or its equivalent, were generally considered to be an English bilingual. Therefore, we applied the adapted Language Experience and Proficiency Questionnaire (LEAP-Q) to fully describe participants’ bilingual learning experience and contextualize their valid Mandarin–English bilingual status with the Language Background Questionnaire. All of the twenty-one subjects qualified as a Mandarin–English bilingual person.

#### 2.2.2. Working Memory Measurement (Reading Span Task)

Reading span tasks in both Mandarin and English were taken by 21 participants after the fMRI scan. According to Daneman and Carpenter’s [33] classic reading span task (RST) and listening span task (LST) findings, the RST is significantly correlated with the LST, and span tasks are a powerful predictor of the neural bases of individual differences in verbal working memory capacity. In the current study, the reason that the RST was selected for the experiment, rather than the LST, was due to the fact that sentence comprehension demands the extensive storage of partial and final products in the service of complex information processing, as well as requiring maximum attentional control [101]. Another reason is that the RST is excellent for testing parallel control functions in executive systems of working memory. In addition, the way the RST is administered, with participants reading sentences for judgement, is much more cognitively and neurally correlated with the fMRI context than the LST. In this study, the RST was performed in two different versions—in Mandarin and English—and was measured online in an adaptation of Conway et al.’s design [99] (http://www.pitt.edu/~tol7/res/research/psych-tests/rspan/, accessed on 7 May 2016), to indicate the significant reliability of working memory tests.

During the RST procedure, subjects were required to remember two, three, four, five, or six alphabets while completing sentence semantic judgement tasks. That is, for each trial, two, three, four, five, or six alphabets would present, equally, three times in five blocks. Thus, there were 66 sentences. Another set of three two-alphabet trials in one block were used as practice trials.

#### 2.2.3. Nelson–Denny Reading Test

The Nelson–Denny Reading Test [100] provides measures of comprehension, reading rate and vocabulary. It is widely used in research studies with undergraduate students and for assessment purposes in the USA. Two parts of the test were conducted in the current study: the reading comprehension test (7 passages with 38 questions) and the vocabulary test (80 questions mainly on vocabulary semantic meaning and pragmatic use) were performed by the participants.

### 2.3. Experimental Stimuli

The stimuli for the fMRI valence task were 63 English dialogic sentence sets with equal amounts of 21 literal, metaphorical, and metonymic dialogic sentence sets in English. Literal, metaphorical, and metonymic stimuli were all controlled in each task. Circumstantial metonymy is less contextualized than systematic metonymy. Hence, we assume that participants process circumstantial metonymy longer than systematic metonymy because of the familiarity shown in metaphorical comprehension [102,103,104,105,106,107,108,109].

A valence task was selected for several reasons. As Rapp et al. [13] proposed, the valence task may require “deep semantic processing and an assessment of the ‘ground’ of the metaphor”. It should be noted that semantic relations among words are important for valence decisions because figurative meaning has to be integrated based on the analysis of the semantic relations of words. Besides, research [107,108]) has demonstrated that valence judgment and semantic relatedness judgment in metaphor processing require the processing of figurative meaning and semantic relations. In the valence judgment task, participants decide whether sentences have a positive, neutral, or negative connotation by comprehending the sentences in each trial. The valence judgement was normalized and balanced in relation to the stimuli (half positive, half negative) for each condition.

### 2.4. General Experimental Procedure

Subjects were given instructions and practice trials prior to performing the event-related design. There were two types of sentences in each trial: a context sentence and a target sentence. They were sectioned from a string of relevant pragmatic, daily, colloquial dialogue by different interlocutors or two logical narrative sentences. The context sentence was followed by the target sentence. During the valence task, in which subjects read and made a valence judgement within six seconds, they were asked to read each presented target sentence in the mind and decide, as fast and as accurately as possible, whether this condition (context plus target sentence) had a positive, negative, or neutral meaning, indicating their decision by pressing one of three buttons after reading the context sentence. The left index finger responded to negative meaning, the right index finger responded to positive meaning, and the right middle finger responded to neutral connotation. Complete sentences were visually presented via a mirror mounted above the head coil within the scanner. Each sentence was shown on one line in silver–white letters against a black background. The task was implemented in 3 runs, with each run containing 21 stimuli of 3 intermixed conditions (LIT, META, METO). For instance, in the literal condition, the context sentence “One Emergency Room nurse says to another:” was followed by the target sentence for another 6 s, during which the participants made a valence judgement: “The patient in room 17B says she needs another pain pill.” In the metaphor condition, the context could be “At a restaurant, a server says to a cook:” and the following target sentence: “The fat pig on table 7 says he doesn’t have enough eggs.” In the metonymy condition, the context was provided as: “In a bar, an old bartender says to the new hire:”, and then, the target sentence followed: “The martini at table 10 is a regular customer.” During the inter-stimulus intervals, a fixation cross was displayed on the screen for 6 s.

The number of positive-valenced and negative-valenced sentences was balanced in all conditions. Each condition had an equal number of high- and low-concrete, familiar, and imageability sentences. Each inter-stimulus interval was 6 s. During the inter-stimulus intervals, a fixation cross was shown on the screen. Subjects’ responses and reaction times (RTs) were recorded with E-Prime 2.0 software (www.pstnet.com/e-prime/, accessed on 4 July 2016).

**Figure 1:**
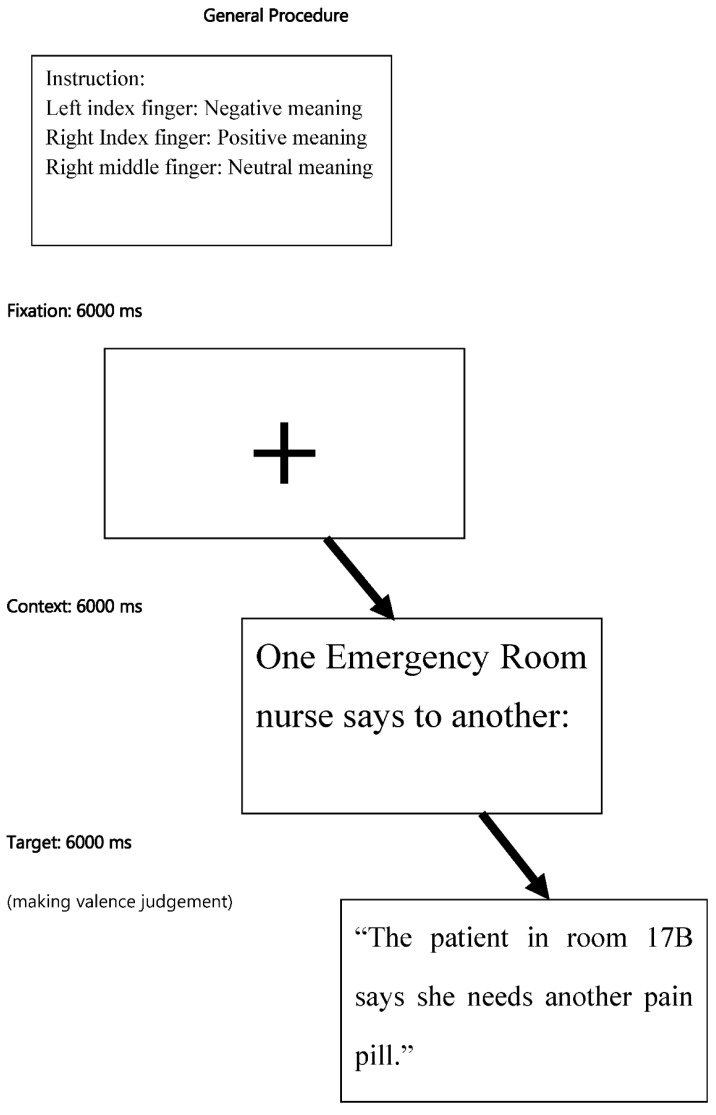
Experimental procedure of the fMRI valence task.

After undertaking the valence judgement tasks [10,13] inside the scanner, participants completed The Language Experience and Proficiency Questionnaire (LEAP-Q) and Nelson–Denny Reading Test [100] outside the scanner. Afterwards, participants were asked to finish the reading span task (RST) in both Mandarin [109] and in English [33,99] online within one week (Figure 1).

## 3. Results

### 3.1. Data Acquisition

#### 3.1.1. Behavioral Data Acquisition

Subjects’ responses and RTs were recorded with E-Prime 2.0 software (www.pstnet.com/e-prime/, accessed on 4 July 2016).

#### 3.1.2. Imaging Data Acquisition

Brain imaging data were acquired using a 3T Siemens Magnetom Trio, A Tim System scanner (Siemens Healthcare, Erlangen, Germany) equipped with a 12-channel standard head coil. Functional images were acquired with an echo-planar image sequence sensitive to BOLD-contrast (TE 30MS, TR 2.5 s, αflip angle 70°). The volume covered the whole brain with a 64 × 64 matrix and 46 transverse slices (4 mm thickness with a 0 mm inter-slice gap; voxel size 3.00 × 3.00 × 3.00 mm). High-resolution MPRAGE 3D T1-weighted scans were also acquired for anatomical localization. Participants lay supine in the scanner where they could directly hear and respond verbally with the vocal transceiver in the scanning room from where instructions and auditory cues were delivered. Behavioral responses (left index finger for responding to negative meaning; right index finger for positive meaning) were given via an MRI-compatible response (Luminex platform, Luminex Corp, Austin, TX) pad connected to a computer that logged reaction times. The same computer ran with E-Prime 2.0 software (www.pstnet.com/e-prime/, accessed on 4 July 2016). Head motion was minimized using foam padding on the sides of the head. Right before the start of the experimental sessions, T2-weighted anatomical images were acquired in the same plane as the functional images using a turbo spin echo sequence (TR = 2500 ms, TE = 30 ms, FA = 90 u, FOV = 1,926,192 mm, matrix size = 2,566,256, in-plane resolution 0.7560.75 mm). In each experimental session, 152 whole-brain echo-planar functional images were acquired in 33 contiguous 4 mm axial slices parallel to the AC-PC line (TR = 2500 ms, TE = 30 ms, FA = 80 u, FOV = 1,926,192 mm, matrix size = 64,664, in-plane resolution 363 mm). Before analyses, the first three scans of each session were discarded to account for the magnetic saturation effects. Whole-brain T1-weighted anatomical images (1 mm^3^) were also acquired prior to the experiments.

#### 3.1.3. Imaging Data Preprocessing

The resulting functional scans were preprocessed using Matlab (version R2016a, Mathworks, Inc., Natick, MA, USA) and SPM 8 (Wellcome Trust Centre for Neuroimaging, UK, http://www.fil.ion.ucl.ac.uk/spm/software/spm8, accessed on 8 February 2017). Slice timing correction was performed, using the first slice as a reference, followed by the realignment and adjustment of head motion using the first image of each session as a reference after realigning the first image of each session to the first image of the first session (no subjects moved more than the length of a voxel in any one direction; thus, none were excluded from the analysis); functional and anatomical images were co-registered using a two-step procedure involving the participant’s T2- and T1-weighted anatomical images. Functional images were spatially normalized to the standard stereotaxic Montreal Neurological Institute (MNI) space by applying the transformation matrix, derived by normalizing the T1-weighted anatomical image to the SPM 8 templates/T1.nii image.

### 3.2. Data Analysis

#### 3.2.1. Behavioral Data Analysis

Behavioral data collected during scans were averaged across each condition following logarithmic transformation to account for the reaction time (RT) outliers (Ratcliff, 1993 [110]). Results across the three conditions (LIT, METO, META) were compared using one-way analyses of variance (ANOVAs).

#### 3.2.2. Behavioral Data Results

Mean score of the reading comprehension was 21.62 (total score: 38.00; SD = 6.80), and the mean score of vocabulary was 35.50 (total score: 80.00; SD = 12.09) from The Nelson–Denny Reading Test [100]. English working memory measurement mean score was 0.7608 (total score: 1.0000; SD = 0.1161) and Mandarin working memory measurement mean score was 0.7675 (total score: 1.000; SD = 0.1430). The Pearson correlation between the English WM and Mandarin WM score was 0.870 (R^2^ = 0.757). This illustrates that, in Mandarin–English bilingual minds, English WMC is strongly correlated with Mandarin Chinese WMC, which validates our research in studying the role of bilingualism in English and Mandarin speakers.

For the behavioral data, we constructed linear mixed models using the lme4 package [111] in R version 3.3 (R Core Team, 2016). Language type (literal, metonymy, and metaphor) was included as a fixed effect, and random factors included intercepts for participants and items. Table 1 reported the mean reaction time (RT) of each language type and its standard error. The reaction time (RT) among literal, metaphor, and metonymy comprehension slightly varied. Individuals spent the most time processing metonymy and the least on literal sentences; however, to our surprise, the Type II Wald F test with Kenward–Roger df showed no significant effect of language type (F < 1, *p* = 0.59) (Table 1). In consequence, we assume that the level of familiarity or difficulty for these three types of language will differ, as fMRI evidence has revealed.

To explore our speculation that items in the systematic literal and metonymies were unfamiliar (more difficult) to our subjects, we constructed another mixed effect model with language type (literal and metonymy) and subtype (circumstantial and systematic) as fixed effects, as well as random participants and items intercepts. The Type II Wald F test with Kenward–Roger df showed a near significant effect of subtype (F (1, 36.94) = 3.03, *p* = 0.08), with mean reaction time in the systematic subtypes (M = 3978.72; SE = 151.78) longer than that in the circumstantial subtypes (M = 3734.06; SE = 146.82), *p* = 0.08. Neither the main effect of language type nor an interaction between the two were discovered (both Fs < 1). This confirmed our speculation to such a degree that we further conducted a re-analysis of the fMRI data, focusing on systematic and circumstantial view analyses of the trials.

#### 3.2.3. Imaging Data Analysis

All image processing operations and analyses were performed with Matlab (version R2016a, Mathworks, Inc., Natick, MA, USA), and d SPM 8 (Wellcome Trust Centre for Neuroimaging, UK, http://www.fil.ion.ucl.ac.uk/spm/software/spm8, accessed on 8 February 2017). The functional images of each participant were corrected for motion and realigned in the first stage of data analysis. T1 anatomical images were co-registered to the mean of the functional data and normalized with the MNI (Montreal Neurosciences Institute) 152 template. Finally, the functional images were smoothed. Model time courses were calculated by defining stimulus onset asynchrony from the protocol using a convolved with the canonical SPM hemodynamic response function (HRF) to specify the design matrix. Condition and participant effects were estimated according to the general linear model (GLM) in each voxel.

#### 3.2.4. General Linear Model Analysis

At the individual level, brain activity during the execution of the valence task was estimated on a voxel-by-voxel basis using the GLM implemented in SPM 8. The GLM had two regressors of interest in this study: vocabulary scores and working memory capacity scores. In the first level processing analysis, regressors of no interest were the six parameters describing head motion plus the constant regressors accounting for the mean session effect. Linear contrast images were generated for each participant using pairwise comparisons between tasks or between the task and the implicit baseline. The participant-specific contrast images of parameter estimates were used as inputs to a random effects model to permit group-level inferences. The resulting statistical maps were submitted to a voxel-level threshold of *p*-value < 0.001, uncorrected, and a cluster extent threshold of *p*-value < 0.05, corrected, for the whole brain. The cluster extent thresholds were determined for each group-level analysis using the function CorrClusTh.m (v. 1.12) written in Matlab by Thomas Nichols (“CorrClusTh.m” could be found on http://www2.warwick.ac.uk/fac/sci/statistics/staff/academic-research/nichols/scripts/spm, accessed on 8 February 2017), and were in the range of 196 to 270 voxels.

For the two variables of interest in the second-level analysis, we constructed two regression models, respectively: one with vocabulary size as a regressor of interest and working memory as a controlled covariate; the other model with working memory capacity as a regressor of interest and vocabulary size as a controlled covariate [70].

### 3.3. Metaphor and Metonymy in General Processing

#### 3.3.1. META > METO

In the contrast of META > METO, surprisingly, we did not find a significant difference based on voxel analysis in our Mandarin–English bilinguals. However, understanding how figurative expressions pose a challenge for the overall language processing mechanism is vital. Previous research has presented that metaphorical conceptualizations might be stored as fixed neural circuits in the brain that are automatically activated when processing metaphors [112]. In an fMRI study, Gallagher and colleagues [113] found support for a connection between metaphor comprehension and ToM (theory of mind) since both tasks activate the medial prefrontal cortex. Conversely, others have found evidence implying that the development of language skills precedes the development of ToM, thus reversing the link [114].

To process an unfamiliar figurative expression, increasing the engagement of the RH in order to compute more complex semantic information has been reported in Giora, Zaidel, Soroker, Batori, and Kasher’s research [106]. They found differential effects in the right and left brain lesions during the understanding of salient metaphors. Few bilingual studies have addressed the RH neural efficiency in different non-native languages, and it seems that cerebral asymmetries are disclosed in bilingual figurative processing despite the fact that they are not entirely consistent with native speaker processing patterns [115].

#### 3.3.2. METO > META

**Figure 2:**
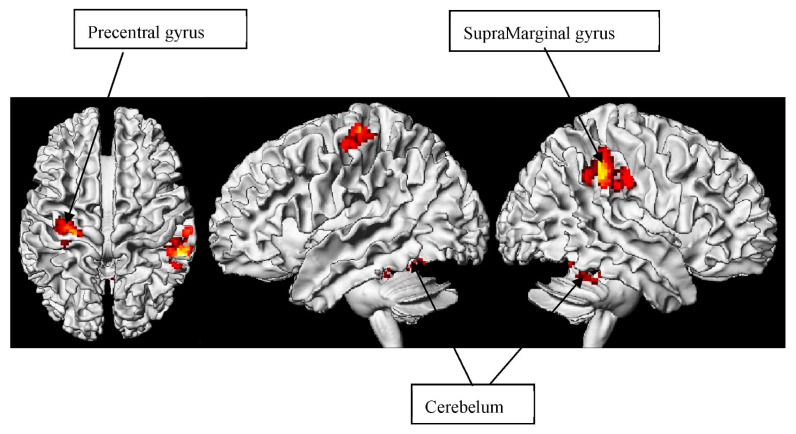
Differential contrast for metonymy > metaphor contrast.

The contrast of METO > META in our study, which has not yet been compared in other studies, suggests the primary involvement of the SupraMarginal, cerebellum, and precentral gyrus regions, as displayed in Table 2 and Figure 2. We have previously defined metonymy as the notion of referential dependency and functional correspondence. Distinct from metaphor, metonymy not only requires an individuals’ pragmatic adjustment when seeking the source of the target concept for functional correspondence, but also the perceptual integration of reference transfer (e.g., in the form of producer-for-product). Individuals need to be more sensitive to the semantic and imagery clues beyond the metonymy structure than when processing metaphor. This is reflected in the role of the right SupraMarginal for making phonological word choices, as well as inhibiting and projecting emotions, rather than being egocentric [116]. Here, we speculate that metonymy comprehension may appear to be associated with an individual’s social perspectives.

This speculation is supported by our cerebellum evidence. As formerly mentioned, we have pinpointed cerebellum activation to attention, phonological, and semantic activity. Essentially, the role of the cerebellum is empirically related to its role in linguistics [117], as well as cognitive and behavioral-affective functions [118], and registered as the “cognitive cerebellum” [119,120]. Higher-level language and metalinguistic abilities in figurative tasks have also been investigated in relation to cerebellar lesions [121,122]. For example, Murdoch and Whelan [123] described 10 patients with primary left cerebellar strokes who had difficulties in producing multiple definitions (provision of two distinct meanings of spoken homophonic words) and recreating sentences in figurative and ambiguous language tests, in word association tasks, in antonym/synonym generation, and in interpreting semantic absurdities.

While research into the lesions of these regions has identified the cerebellum’s linguistic role, our findings may provide new insights into the neural connection between the cerebellum and different figurative language types. Therefore, in line with the recent studies in cerebellar theory related to reading, our findings further strengthen the link between the cerebellum and figurative language processing. In addition, the precentral gyrus has been implicated in mental imagery strategies and episodic memory retrieval [124], which are relevant to metonymy.

#### 3.3.3. Metaphor and Circumstantial Metonymy and Systematic Metonymy Processing

In order to fulfill our research questions further, we conducted another whole-brain correlation analysis. We distinguished our stimuli with three conditions (literal, metaphor, and metonymy) into “systematic” and “circumstantial” views and, thus, into five subtypes: systematic literal, circumstantial literal, systematic metonymy, circumstantial metonymy, and metaphor contrasts.

#### 3.3.4. Vocabulary Effects

##### META > C LIT

**Figure 3:**
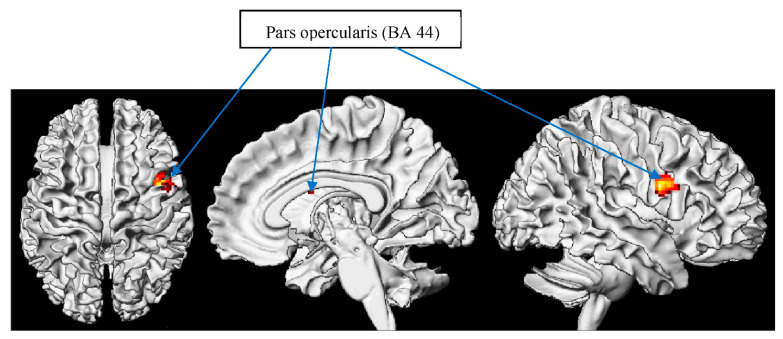
Vocabulary effect of metaphor > circumstantial literal.

In Table 3 and Figure 3, it can be seen that the vocabulary effect of metaphor relative to circumstantial literal contrast was, not surprisingly, found in IFG. However, interestingly, it was found in the right hemisphere rather than in the typical left hemisphere. Distinct from the RH spillover hypothesis that increases RH activation due to a higher ratio of processing demands in lower capacity individuals, our study found that individuals with a higher vocabulary size, in fact, depend more on their RH. In other words, when comprehending metaphor, in contrast to literal reading, our subjects with lower vocabulary sizes were dominantly LH-reliant, without resorting to RH neural resources. However, for subjects with higher vocabulary sizes, they apparently recruited much greater neural reserves from RH. Although no general advantages for figurative language processing in the RH have been claimed, Bohrn et al. [125] propose that the RH is involved with novel metaphors and semantic processes. Hence, it may be explainable that, in metaphor processing, individuals with higher vocabulary sizes recruit their RH for conceptualization strategies through visual imagery [126] and “imagery thinking” [127] in a flexible way.

##### META > C METO

**Figure 4:**
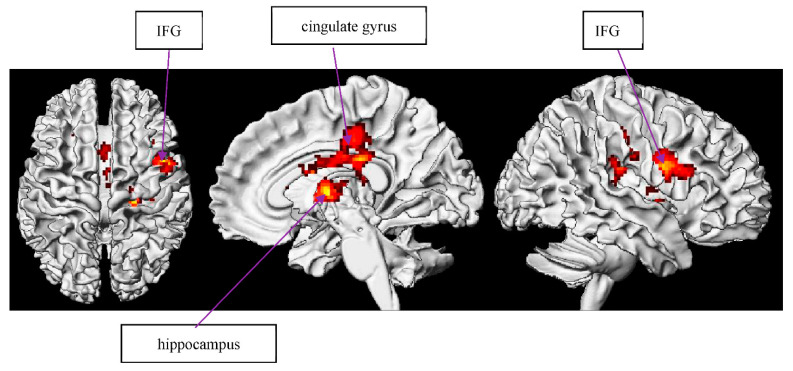
Vocabulary effect of metaphor > circumstantial metonymy.

This contrast presented in Table 4 and Figure 4 may not have been investigated in previous research. In parallel, results have been found in the same regions but different hemispheres. Compared with the current study, in accordance to our META > C LIT contrast, in this META > C METO analysis, the right IFG was revealed for robust activation in individuals with higher vocabulary sizes during the processing of metaphors. This is consistent with the prior research of Rapp et al.’s meta-analysis [128], suggesting that the overall metaphor contrast shown is activated, mostly, in the IFG in the LH, while our contrast was found in the RH.

##### C METO

Our results of large right-activated regions in the superior temporal gyrus (STG) and posterior cingulate cortex are in line with RH involvement with increased demands [107,129] and with deceased individual reading skills [18,71,130,131]. Prat et al. propose “neural efficiency” suggesting that, when comprehending metaphor, individuals with a lower vocabulary size involve more RH assistance, whereas individuals with higher vocabulary size do not [71,131]. Another study posited that LH efficiency is pertinent to English–Spanish bilingual metaphor understanding [132], illustrating that the LH is more sensitive than the RH to metaphor familiarity. Particularly, we found that, when comprehending metonymy, individuals with a lower vocabulary size also recruit more RH neural resources. The possible reason for increased RH involvement with increased processing demands is that this function implies a greater need for more general cognitive processes, such as response selection and/or inhibition [133]. The phenomena which the RH inferior frontal area activates is also the phenomena that suggests that dominant interpretation needs to be suppressed to accurately comprehend critical utterance as a metonymy item. Additionally, bilateral activations of the hippocampus and cingulate gyrus reflect that individuals with lower vocabulary sizes may resort to more episodic memory in order to perceive metonymic sentential context from their vocabulary relevance. There are individual differences in retrieving extra RH employment for neural resources and demonstrating bilateral engagement for relevant cognitive representations between people with larger and smaller lexicons.

#### 3.3.5. Working Memory Capacity Effects

The role of working memory capacity is primarily significant in metonymic processing and is highlighted under both circumstantial metonymy and circumstantial literal comprehension.

##### C METO > C LIT

**Figure 5:**
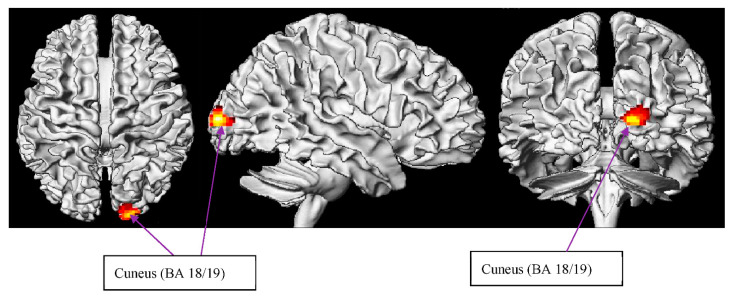
WM effect of circumstantial metonymy > circumstantial literal.

Remarkably, the cuneus has been found to be mainly evoked in the RH. In addition to the traditional role as a site for fundamental visual processing, gray matter volume in the cuneus is associated with better inhibitory control [134]. Bilateral activation has been indicated in the cuneus during WM tasks in fMRI studies [135,136], revealing that stronger cuneus activation is reported for high-workload conditions [135]. In a recent electroencephalography (EEG) study, Haldane et al. [134] proposed that cuneus increases its activation with increased alpha activity, reflecting active functional task-irrelevant inhibition. This is in tandem with the resource-sharing theory [33,47,137] related to the working memory model. Based on the theory, there is a trade-off between information manipulation and storage. The more efficiently the individuals store their critical information, the more available resources they can infer from. Besides, individuals with higher-span scores are likely to possess more robust knowledge about the distribution of syntactic constructions [138,139]. Accordingly, we can infer that individuals with higher working memory capacity scores are not only better at storing critical items in a metonymic sentential context but also at manipulating critical resources for the target sentence in the experiment by diminishing redundant information. Thus, we strengthen the proof that the cuneus, as an inhibitory control, tends to contribute to irrelevant information inhibition for metonymy processing (Table 5 and Figure 5).

##### C METO

**Figure 6:**
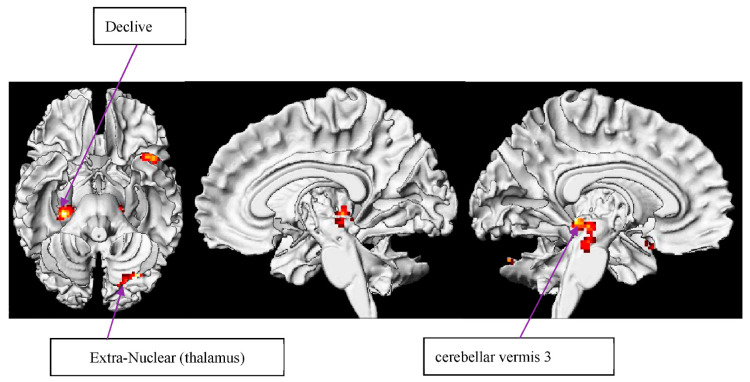
WM effect of circumstantial metonymy baseline.

Evidently, the cerebellum was prominent in the correlation between working memory capacity and circumstantial metonymy condition in our study (Table 6 and Figure 6). In the same vein, we support the associations between working memory and the cerebellum [140] as well as the investigation that the cerebellum is active during working memory tasks [141,142]. The activation of the cingulate gyrus was consistent with Osaka et al.’s finding [143] in a higher-span group and may, again, support our speculation that individuals with better working memory capacity (the higher-span group) resort to a perspective-taking strategy for comprehending a broader sense of metonymic connotation. Notably, the observation of elevated BOLD responses in the lentiform nucleus (where putamen is contained) can be inferred as the putamen’s role and is responsible for inhibitory function, especially for irrelevant information during working memory execution [144], which also supports our previous finding in relation to cuneus function. It is therefore indicated that individuals with better working memory capacity also recruit another strategy to better manipulate their limited WM capacity to focus on the target critical items. Additionally, a lentiform nucleus dependent on working memory has been found in rule-based tasks via hypothesis testing [145].

## 4. Discussion

In this study, similar fronto-temporal networks for figurative language processing patterns were found: the superior temporal gyrus (STG) and the inferior parietal junction (IPJ) for metaphor and metonymy comprehension, respectively. The STG was activated in metaphorical comprehension, as is also discussed in the previous literature [146]. The IPJ was engaged in metonymic processing, which was different from the previous findings for metaphor tasks [147]. We assume that processing metonymy requires different neuro-cognitive resources compared to metaphor understanding. This theory was demonstrated, for example, in the cuneus when reading circumstantial metonymies. Bialystok and Luk [61] indicate that inhibitory control modulates WM in bilingualism. In what follows, our study has illustrated that the cuneus, where putamen is contained in relation to WM, is activated in higher-span bilinguals. It is suggested that Mandarin–English bilinguals with better WM and vocabulary size resort to inhibitory control use, filtering out irrelevant information in metonymy comprehension. Moreover, cingulate gyrus activation was also revealed in better WMC bilinguals; they demonstrated mental flexibility to adopt the subjective perspective of critical figurative items using self-control.

We found out that the described neural correlates link directly to metaphor and metonymy processing, as well as the effects of WM and vocabulary size. Our first hypothesis was supported by the metaphorical processing demonstrated, which linked some important areas of the brain with metonymic processing. Our results are also consistent with the cognitive and linguistic activities of cerebellar engagement in metonymy interpretation. The second and third hypotheses were partially supported by the whole-brain volumetric analysis, but the effects of WM and vocabulary varied. The only WM effect that this study has proven is circumstantial metonymy contrast. We did not find neural correlation between working memory capacity and metaphor comprehension, as presented in Chiappe and Chiappe’s inquiry [77]. However, the activation of the middle frontal gyrus (MFG) was consistent with its association with working memory [148] and inhibitory control in bilingualism [61,69,149,150]. In terms of the effects of vocabulary size, interestingly, the finding of metonymic processing resonated with Prat, Manson, and Just’s metaphorical investigation [70]. Our Mandarin–English bilinguals with lower vocabulary size also spilled into their RH for compensatory resources as a neural efficiency strategy in metonymical processing. This may reflect the neuro-cognitive similarity between metaphor and metonymy and the need for bilateral semantic networks for learners with smaller vocabularies, even in bilinguals. Hence, circumstantial metonymy is neurally correlated with a smaller lexicon in bilinguals.

No neural correlations were found between systematic metonymy, the effects of WM, or the size of the lexicon. According to the Graded Salience Hypothesis [102,103,104,105,106]), whether a word is used in its literal or figurative meaning is far less relevant than whether that word and its intended meaning are familiar/lexicalized and salient in the context in which it occurs. That is, GSH predicts that the performance of lexicalized metaphors will be better than novel ones. As a result, our findings may extend GSH to the implication that metonymical processing is of a similar trend. Whether the sentences are conveyed in metaphorical or metonymical conditions, individuals comprehend circumstantial conditions which are more familiar, lexicalized, and less difficult, even for bilinguals. This was observed with the Mandarin–English bilinguals in our study.

Furthermore, as previously unfolded in our literature review, the evidence from psycholinguistic and neurolinguistic studies propose that metonymy comprehension not only involves consideration and the subsequent rejection of a literal sense, but it also often involves the knowledge of cultural conventions and idealized cognitive models, if only at a subconscious level. As a result, beyond the traditional definitions and linguistic structures of both metaphor (as conceptual mapping or lexical pragmatic adjustment processes) and metonymy (in a circumstantial setting with higher conventionalization or a systematic setting with a lesser conventionalization degree) [19], we propose that a person requires the ability to make inferences, take different perspectives for reasoning, and integrate social-cultural cues of the target language to activate the mental imagery network required for both metaphor and metonymy understanding.

Since the awareness of conceptual metaphors does not guarantee bilingual learners’ automatic access to conceptual metaphor and metonymy comprehension, nor their active computation of metaphorical/metonymic mappings [151] during figurative language processing [152], our findings support the neural-imaging evidence. There exists cognitive distance in bilinguals’ minds between relating their (vocabulary) knowledge and experiences to the target metaphorical concepts precisely and schematizing concepts appropriately to full comprehension. In terms of pragmatic and/or sociocultural awareness, bilinguals should be informed of the fact that not every type of figurative language is entirely interpretable. However, as Chen and Lai’s study [153] clearly argues, it is necessary to identify metaphorical mappings to assist L2/EFL learners in connecting context and target domain sentences and to encourage them to associate existing and universal knowledge with unfamiliar and specific knowledge. This may offer widespread implications for second or foreign language acquisition and teaching paradigms.

## 5. Limitations

In our study, several limitations regarding the sample size and individual study parameters should be acknowledged. The first limitation stems from the gender distribution. In light of the gender differences in the human brain structure [154] and in language processing [155], this unequal distribution might have influenced the results we found for both metaphor and metonymy processing. Secondly, due to (1) the nature of fMRI design and (2) the fact that our participants were bilinguals whose native language is Mandarin Chinese, the study may lack generalizability to the larger population, the identification of developmental trajectories, and the distinguishment of causes from effects [156].

## 6. Conclusions

The effects of WM and vocabulary size were found in circumstantial metonymy rather than in metaphor and systematic metonymy comprehension. However, inhibitory control and cerebellar association in bilingualism [61,69,149,150] shown in the middle frontal gyrus (MFG) [148] were supported in working memory modulation. Contrary to our hypothesis, no voxel analysis of systematic metonymy contrast was observed in the vocabulary and working memory effect correlation; however, since previous investigations have proven that task demands may modulate the effects of WMC [157,158,159,160,161], we assume that different types of metonymy (systematic and circumstantial metonymy) may also recruit neural demands at different levels.

Taken together, in our volumetric analysis, both the left posterior PFC (BA 44/9) and the left PFC (BA 47) received activated responses. Linking with the left tempo-frontal network for figurative language processing, these regions have been suggested to robustly connect with the controlled retrieval and selection of information from semantic memory [162]. The corresponding left PFC regions have been proposed to subserve semantic working memory processes [163].

Future research should study the relevance between neural efficiency and different types of metonymies (e.g., systematic and circumstantial metonymy) and how the RH recruits more neural resources to support bilateral computation in dynamic patterns for bilingual speakers with native languages other than English. Our fMRI evidence suggests that it is critical to reinforce the neuro-cognitive and neurolinguistic foundations of metaphor and metonymy reading in bilingualism. Apparently, the area of neural correlation between metaphor and metonymy processing with Mandarin–English bilinguals calls for much more research. This approach promises to provide greater insight into the flexible neural mechanisms of figurative languages and other cognitive abilities for bilingualism.

## Figures and Tables

**Table 1:** Behavioral data of fMRI scanning for three language types.

Type	Mean RT (ms)	Standard Error	*p* (F-Test)
Literal	3830.27	141.36	0.59
Metaphor	3843.44	140.67	
Metonymy	3996.08	143.35	

**Table 2:** Whole brain analysis of metonymy minus metaphor contrast (differential contrast for metonymy > metaphor sentences. Random effects model, *p* < 0.001 uncorrected, *p* < 0.05 FDR-corrected. Coordinates are reported in MNI space.).

Region	BA	Hemisphere	Voxels	X	Y	Z	Max *t*-Value
SupraMarginal gyrus	40	R	733	60	−36	34	5.40
Cerebelum 4_5		R	312	20	−42	−24	5.28
Precentral gyrus	4	L	238	−30	−18	58	5.19

**Table 3:** Vocabulary effect of metaphor minus circumstantial literal contrast. Note: inferior frontal gyrus—IFG; random effects model: *p* < 0.001 uncorrected; *p* < 0.05 FDR-corrected. Coordinates are reported in MNI space.

Region	BA	Hemisphere	Voxels	X	Y	Z	Max *t*-Value
Sub-gyral		R	225	40	4	22	6.34
IFG	6/44/45	R	312	52	0	26	4.56

**Table 4:** Vocabulary effect of metaphor minus circumstantial metonymy contrast. Note: ventral anterior nucleus—VAN; inferior frontal gyrus—IFG; middle temporal frontal—MTG. Random effects model: *p* < 0.001 uncorrected, and *p* < 0.05 FDR-corrected. Coordinates are reported in MNI space.

Region	BA	Hemisphere	Voxels	X	Y	Z	Max *t*-Value
VAN		R/L	781	10	−6	6	8.77
IFG	6/44/45	R	1031	22	−22	44	8.16
MTG	38	L	181	−40	−6	−24	6.45

**Table 5:** WM effect of circumstantial metonymy minus circumstantial literal contrast. Random effects model, *p* < 0.001 uncorrected, and *p* < 0.05 FDR-corrected. Coordinates are reported in MNI space.

Region	BA	Left/Right	Voxels	X	Y	Z	Max *t*-Value
Cuneus	18/19	R	195	20	−100	8	7.01

**Table 6:** WM effect circumstantial metonymy baseline. Note: superior temporal gyrus—STG. Random effects model: *p* < 0.001 uncorrected, and *p* < 0.05 FDR-corrected. Coordinates are reported in MNI space.

Region	BA	Hemisphere	Voxels	X	Y	Z	Max *t*-Value
STG	38	R	102	−44	16	−18	5.51
Declive		L	242	−30	−78	−24	4.86
Extra-Nuclear		R	197	0	−38	−4	5.25
cerebellar vermis 3		L	100	28	−28	−4	9.82

## Data Availability

Data will be available upon request from the corresponding author.

## References

[1] Bischofshausen S., Makoid L.A., Cole J. (1989). Effects of Inference Requirements on Comprehension and Recognition of Metaphors. Metaphor Symb. Act..

[2] Levorato M.C., Cacciari C. (1992). Children’s comprehension and production of idioms: The role of context and familiarity. J. Child Lang..

[3] Gibbs W.W. (1995). Lost science in the third world. Sci. Am..

[4] Makkai A., Boatner M.T., Gates J.E. (1995). A Dictionary of American Idioms.

[5] Milosky L.M. (1994). Nonliteral language abilities: Seeing the forest for the trees. Language Learning Disabilities in School-Age Children and Adolescents: Some Principles and Applications.

[6] Kennedy G.A. (2008). The Art of Rhetoric in the Roman World: 300 BC–AD 300.

[7] McCarthy M., Carter R. (2004). “There’s millions of them”: Hyperbole in everyday conversation. J. Pragmat..

[8] Brownell H.H., Simpson T.L., Bihrle A.M., Potter H.H., Gardner H. (1990). Appreciation of metaphoric alternative word meanings by left and right brain-damaged patients. Neuropsychologia.

[9] Rinaldi M.C., Marangolo P., Baldassari F. (2004). Metaphor processing in right braindamaged patients with visuo-verbal and verbal material: A dissociation (re)considered. Cortex.

[10] Mashal N., Faust M., Hendler T., Jung-Beeman M. (2007). An fMRI investigation of the neural correlates underlying the processing of novel metaphoric expressions. Brain Lang..

[11] Stringaris A.K., Medford N., Giora R., Giampietro V.C., Brammer M.J., David A.S. (2006). How metaphors influence semantic relatedness judgments: The role of the right frontal cortex. NeuroImage.

[12] Lee S.S., Dapretto M. (2006). Metaphorical vs. literal word meanings: fMRI evidence against a selective role of the right hemisphere. NeuroImage.

[13] Rapp A.M., Leube D.T., Erb M., Grodd W., Kircher T. (2004). Neural correlates of metaphor processing. Cogn. Brain Res..

[14] Rapp A.M., Leube D.T., Erb M., Grodd W., Kircher T. (2007). Laterality in metaphor processing: Lack of evidence from functional magnetic resonance imaging for the right hemisphere theory. Brain Lang..

[15] Shibata M., Abe J.-I., Terao A., Miyamoto T. (2007). Neural mechanisms involved in the comprehension of metaphoric and literal sentences: An fMRI study. Brain Res..

[16] Ferstl E.C., Neumann J., Bogler C., Von Cramon D.Y. (2008). The extended language network: A meta-analysis of neuroimaging studies on text comprehension. Hum. Brain Mapp..

[17] Benedek M., Beaty R., Jauk E., Koschutnig K., Fink A., Silvia P.J., Dunst B., Neubauer A.C. (2013). Creating metaphors: The neural basis of figurative language production. NeuroImage.

[18] Lai V.T., Desai R.H. (2016). The grounding of temporal metaphors. Cortex.

[19] Piñango M.M., Zhang M., Foster-Hanson E., Negishi M., Lacadie C., Constable R.T. (2017). Metonymy as referential dependency: Psycholinguistic and neurolinguistic arguments for a unified linguistic treatment. Cogn. Sci..

[20] Michl D. (2019). Metonymies are more literal than metaphors: Evidence from ratings of German idioms. Lang. Cogn..

[21] Goossens L. (1995). From Three Respectable Horses’ Mouths: Metonymy and Conventionalization in a Diachronically Differentiated Data Base.

[22] Taylor J.R. (1995). Linguistic Categorization. Prototypes in Linguistic Theory.

[23] Annaz D., Van Herwegen J., Thomas M., Fishman R., Karmiloff-Smith A., Rundblad G. (2009). Comprehension of metaphor and metonymy in children with Williams syndrome. Int. J. Lang. Commun. Disord..

[24] Klepousniotou E. (2002). The Processing of Lexical Ambiguity: Homonymy and Polysemy in the Mental Lexicon. Brain Lang..

[25] Rundblad G., Annaz D., Dimitriou D. (2010). The atypical development of metaphor and metonymy comprehension in children with autism. Autism.

[26] Weiland H., Bambini V., Schumacher P.B. (2014). The role of literal meaning in figurative language comprehension: Evidence from masked priming ERP. Front. Hum. Neurosci..

[27] Lakoff G., Turner M. (1989). More Than Cool Reason: A Field Guide to Poetic Metaphor.

[28] Sweetser E. (1990). From Etymology to Pragmatics: Metaphorical and Cultural Aspects of Semantic Structure.

[29] Qualls C.D., Bodle H., O’Brien R., Treaster B., Blood G.W., Hammer C.S. Idioms in Rural 5th Graders: Effects of Differential Language Exposure. Proceedings of the Annual Convention of the American Speech-Language and Hearing Association.

[30] Qualls C.D., Harris J.L. (2003). Age, Working Memory, Figurative Language Type, and Reading Ability. Am. J. Speech-Lang. Pathol..

[31] Zelinski E.M., Hyde J.C. (1996). Old Words, New Meanings: Aging and Sense Creation. J. Mem. Lang..

[32] Levorato M.C., Cacciari C. (1995). The effects of different tasks on the comprehension and production of idioms in children. J. Exp. Child Psychol..

[33] Daneman M., Carpenter P.A. (1980). Individual differences in working memory and reading. J. Verbal Learn. Verbal Behav..

[34] Salthouse T.A. (1990). Working memory as a processing resource in cognitive aging. Dev. Rev..

[35] Searle J.R., Willis Y.S. (1995). The Construction of Social Reality.

[36] Fraser G. (1995). The Pornographic Imagination in All Strange Away. MFS Mod. Fict. Stud..

[37] Clark H.H., Gerrig R.J. (1983). Understanding old words with new meanings. J. Verbal Learn. Verbal Behav..

[38] Fregni S., Heidlmayr K., Weber K., Peeters D. (2019). An Exploratory fMRI Study on Metonymy and Metaphor Processing. Master’s Thesis.

[39] Hauptman M., Blank I., Fedorenko E. (2022). Non-literal language processing is jointly supported by the language and Theory of Mind networks: Evidence from a novel meta-analytic fMRI approach. bioRxiv.

[40] Rapp A.M., Wild B. (2011). Nonliteral language in Alzheimer dementia: A review. J. Int. Neuropsychol. Soc..

[41] Gregory M.E., Waggoner J.E. (1996). Factors that influence metaphor comprehension skills in adulthood. Exp. Aging Res..

[42] Hasher L., Zacks R.T. (1988). Working memory, comprehension, and aging: A review and a new view. Psychol. Learn. Motiv..

[43] Gildea P., Glucksberg S. (1983). On understanding metaphor: The role of context. J. Verbal Learn. Verbal Behav..

[44] Glucksberg S., Gildea P., Bookin H.B. (1982). On understanding nonliteral speech: Can people ignore metaphors?. J. Verbal Learn. Verbal Behav..

[45] Tourangeau R., Sternberg R.J. (1981). Aptness in metaphor. Cogn. Psychol..

[46] Baddeley A.D., Hitch G. (1974). Working memory. Psychology of Learning and Motivation.

[47] Just M.A., Carpenter P.A. (1992). A capacity theory of comprehension: Individual differences in working memory. Psychol. Rev..

[48] Baddeley A. (2000). The episodic buffer: A new component of working memory?. Trends Cogn. Sci..

[49] Metcalfe J., Shimamura A.P. (1994). Metacognition: Knowing about Knowing.

[50] Baddeley A.D. (1986). Working Memory.

[51] Linck J.A., Osthus P., Koeth J.T., Bunting M.F. (2014). Working memory and second language comprehension and production: A meta-analysis. Psychon. Bull. Rev..

[52] Grundy J.G., Anderson J.A., Bialystok E. (2017). Bilinguals have more complex EEG brain signals in occipital regions than monolinguals. NeuroImage.

[53] DeLuca V., Rothman J., Bialystok E., Pliatsikas C. (2019). Redefining bilingualism as a spectrum of experiences that differentially affects brain structure and function. Proc. Natl. Acad. Sci. USA.

[54] Pigott S., Milner B. (1994). Capacity of visual short-term memory after unilateral frontal or anterior temporal-lobe resection. Neuropsychologia.

[55] D’Esposito M., Detre J.A., Alsop D., Shin R.K., Atlas S., Grossman M. (1995). The neural basis of the central executive system of working memory. Nature.

[56] Salmon E., Van der Linden M., Collette F., Delfiore G., Maquet P., Degueldre C., Maquet P., Degueldre C., Luxen A., Franck G. (1996). Regional brain activity during working memory tasks. Brain.

[57] Na D.G., Ryu J.W., Byun H.S., Choi D.S., Lee E.J., Chung W.I., Cho J.M., Han B.K. (2000). Functional MR Imaging of Working Memory in the Human Brain. Korean J. Radiol..

[58] Lewis S.J., Cools R., Robbins T., Dove A., Barker R.A., Owen A.M. (2003). Using executive heterogeneity to explore the nature of working memory deficits in Parkinson’s disease. Neuropsychologia.

[59] Monetta L., Pell M.D. (2007). Effects of verbal working memory deficits on metaphor comprehension in patients with Parkinson’s disease. Brain Lang..

[60] Bialystok E., Luk G., Peets K.F., Sujin Y.A.N.G. (2010). Receptive vocabulary differences in monolingual and bilingual children. Biling. Lang. Cogn..

[61] Bialystok E., Luk G. (2011). Receptive vocabulary differences in monolingual and bilingual adults. Biling. Lang. Cogn..

[62] Prat C.S., Mason R.A., Just M.A. (2011). Individual differences in the neural basis of causal inferencing. Brain Lang..

[63] Guo T., Liu H., Misra M., Kroll J.F. (2011). Local and global inhibition in bilingual word production: fMRI evidence from Chinese–English bilinguals. NeuroImage.

[64] Martin-Rhee M.M., Bialystok E. (2008). The development of two types of inhibitory control in monolingual and bilingual children. Biling. Lang. Cogn..

[65] Grainger J., Beauvillain C. (1987). Language blocking and lexical access in bilinguals. Q. J. Exp. Psychol. Sect. A.

[66] Brysbaert M. (1998). Word recognition in bilinguals: Evidence against the existence of two separate lexicons. Psychol. Belg..

[67] Dijkstra T., Kroll J.F. (2005). Bilingual visual word recognition and lexical access. Handbook of Bilingualism: Psycholinguistic Approaches.

[68] Dunn L., Dunn L. (1997). Peabody Picture Vocabulary Test.

[69] Bialystok E., Craik F.I., Luk G. (2012). Bilingualism: Consequences for mind and brain. Trends Cogn. Sci..

[70] Prat C.S., Mason R.A., Just M.A. (2012). An fMRI investigation of analogical mapping in metaphor comprehension: The influence of context and individual cognitive capacities on processing demands. J. Exp. Psychol. Learn. Mem. Cogn..

[71] Prat C.S., Keller T.A., Just M.A. (2007). Individual Differences in Sentence Comprehension: A Functional Magnetic Resonance Imaging Investigation of Syntactic and Lexical Processing Demands. J. Cogn. Neurosci..

[72] Kazmerski V.A., Blasko D.G., Dessalegn B.G. (2003). ERP and behavioral evidence of individual differences in metaphor comprehension. Mem. Cogn..

[73] Rundblad G., Annaz D. (2010). Development of metaphor and metonymy comprehension: Receptive vocabulary and conceptual knowledge. Br. J. Dev. Psychol..

[74] Boers F. (2000). Metaphor awareness and vocabulary retention. Appl. Linguist..

[75] Kintsch W. (2001). Predication. Cogn. Sci..

[76] Landauer T.K. (1998). Learning and representing verbal meaning: The latent semantic analysis theory. Curr. Dir. Psychol. Sci..

[77] Chiappe D.L., Chiappe P. (2007). The role of working memory in metaphor production and comprehension. J. Mem. Lang..

[78] Stein-Smith K. (2016). The Role of Multilingualism in Effectively Addressing Global Issues: The Sustainable Development Goals and Beyond. Theory Pract. Lang. Stud..

[79] Amaro J.C., Flynn S., Rothman J. (2012). Third Language Acquisition in Adulthood.

[80] Rothman J., Niño Murcia M. (2008). Multilingualism and identity. Bilingualism and Identity: Spanish at the Crossroads with Other Languages.

[81] Rothman J., Halloran B. (2013). Formal linguistic approaches to L3/Ln acquisition: A focus on morphosyntactic transfer in adult multilingualism. Annu. Rev. Appl. Linguist..

[82] Slabakovaa R., Amarob J.C., Kangc S.K. (2013). L2 regular and novel metonymy: How to curl up with a good Agatha Christie in your L2. Proceedings of the 37th Boston University Conference on Language Development.

[83] Apresjan J.D. (1974). Regular Polysemy. Linguistics.

[84] Eckardt R. (1999). Three ways to create metonymy: A study in locative readings of institution names. Studi Ital. Linguist. Teor. Appl..

[85] Jackendoff R. (1997). The Architecture of the Language Faculty.

[86] Nunberg G. (1979). The non-uniqueness of semantic solutions: Polysemy. Linguist. Philos..

[87] Nunberg G. (1995). Transfers of meaning. J. Semant..

[88] Gibbs R.W. (1994). Figurative Thought and Figurative Language.

[89] Lakoff G., Johnson M. (1980). Conceptual Metaphor in Everyday Language. J. Philos..

[90] Barcelona A. (2000). Metaphor and Metonymy at the Crossroads.

[91] Grice H.P. (1975). Logic and conversation. Speech Acts.

[92] Searle J.R. (1979). The intentionality of intention and action. Inquiry.

[93] Sperber D., Wilson D. (2002). Pragmatics, modularity and mind-reading. Mind Lang..

[94] Chen E., Widick P., Chatterjee A. (2008). Functional–anatomical organization of predicate metaphor processing. Brain Lang..

[95] Wakusawa K., Sugiura M., Sassa Y., Jeong H., Horie K., Sato S., Yokoyama H., Tsuchiya S., Inuma K., Kawashima R. (2007). Comprehension of implicit meanings in social situations involving irony: A functional MRI study. NeuroImage.

[96] Eviatar Z., Just M.A. (2006). Brain correlates of discourse processing: An fMRI investigation of irony and conventional metaphor comprehension. Neuropsychologia.

[97] Uchiyama H.T., Saito D.N., Tanabe H.C., Harada T., Seki A., Ohno K., Koeda T., Sadato N. (2012). Distinction between the literal and intended meanings of sentences: A functional magnetic resonance imaging study of metaphor and sarcasm. Cortex.

[98] Marian V., Blumenfeld H.K., Kaushanskaya M. (2007). The Language Experience and Proficiency Questionnaire (LEAP-Q): Assessing language profiles in bilinguals and multilinguals. J. Speech Lang. Hear. Res..

[99] Conway A.R.A., Kane M.J., Bunting M.F., Hambrick D.Z., Wilhelm O., Engle R.W. (2005). Working memory span tasks: A methodological review and user’s guide. Psychon. Bull. Rev..

[100] Brown J.A., Fishco V.V., Hanna G. (1993). Nelson-Denny Reading Test: Manual for Scoring and Interpretation, Forms G & H.

[101] Engle R.W., Carullo J.J., Collins K.W. (1991). Individual Differences in Working Memory for Comprehension and Following Directions. J. Educ. Res..

[102] Giora R. (1997). Understanding figurative and literal language: The graded salience hypothesis. Cogn. Linguist..

[103] Giora R. (1999). On the priority of salient meanings: Studies of literal and figurative language. J. Pragmat..

[104] Giora R. (2002). Literal vs. figurative language: Different or equal?. J. Pragmat..

[105] Giora R. (2003). On Our Mind: Salience, Context, and Figurative Language.

[106] Giora R., Zaidel E., Soroker N., Batori G., Kasher A. (2000). Differential effects of right-and left-hemisphere damage on understanding sarcasm and metaphor. Metaphor. Symb..

[107] Yang F.G., Edens J., Simpson C., Krawczyk D.C. (2009). Differences in task demands influence the hemispheric lateralization and neural correlates of metaphor. Brain Lang..

[108] Yang F.G., Fuller J., Khodaparast N., Krawczyk D.C. (2010). Figurative language processing after traumatic brain injury in adults: A preliminary study. Neuropsychologia.

[109] Zhou H., Rossi S., Chen B. (2017). Effects of working memory capacity and tasks in processing L2 complex sentence: Evidence from Chinese-English bilinguals. Front. Psychol..

[110] Ratcliff R. (1993). Methods for dealing with reaction time outliers. Psychol. Bull..

[111] Bates D., Maechler M., Bolker B., Walker S., Christensen R.H.B., Singmann H., Dai B., Scheipl F., Grothendieck G. (2011). Package Mixed-Effects Models Using S4 Classes. Package ‘lme4’, R Package Version 1. https://cran.microsoft.com/snapshot/2020-04-13/web/packages/lme4/lme4.pdf.

[112] Lakoff G. (2009). The Neural Theory of Metaphor. https://papers.ssrn.com/sol3/papers.cfm?abstract_id=1437794.

[113] Gallagher H.L., Happé F., Brunswick N., Fletcher P.C., Frith U., Frith C.D. (2000). Reading the mind in cartoons and stories: An fMRI study of ‘theory of mind’in verbal and nonverbal tasks. Neuropsychologia.

[114] Hale C.M., Tager-Flusberg H. (2003). The influence of language on theory of mind: A training study. Dev. Sci..

[115] Cieślicka A.B. (2017). Bilingual figurative language processing. Psychology of Bilingualism.

[116] Silani G., Lamm C., Ruff C., Singer T. (2013). Right Supramarginal Gyrus Is Crucial to Overcome Emotional Egocentricity Bias in Social Judgments. J. Neurosci..

[117] Leiner H.C., Leiner A.L., Dow R.S. (1993). Cognitive and language functions of the human cerebellum. Trends Neurosci..

[118] De Smet H.J., Paquier P., Verhoeven J., Mariën P. (2013). The cerebellum: Its role in language and related cognitive and affective functions. Brain Lang..

[119] Botez-Marquard T., Léveillé J., Botez M. (1994). Neuropsychological Functioning in Unilateral Cerebellar Damage. J. Can. Sci. Neurol..

[120] Gottwald B., Wilde B., Mihajlovic Z., Mehdorn H.M. (2004). Evidence for distinct cognitive deficits after focal cerebellar lesions. J. Neurol. Neurosurg. Psychiatry.

[121] Cook M., Murdoch B., Cahill L., Whelan B. (2004). Higher-level language deficits resulting from left primary cerebellar lesions. Aphasiology.

[122] Whelan B.-M., Murdoch B. (2005). Unravelling subcortical linguistic substrates: Comparison of thalamic versus cerebellar cognitive-linguistic regulation mechanisms. Aphasiology.

[123] Murdoch B.E., Whelan B.-M. (2007). Language Disorders Subsequent to Left Cerebellar Lesions: A Case for Bilateral Cerebellar Involvement in Language?. Folia Phoniatr. Logop..

[124] Cavanna A.E., Trimble M.R. (2006). The precuneus: A review of its functional anatomy and behavioural correlates. Brain.

[125] Bohrn I.C., Altmann U., Jacobs A.M. (2012). Looking at the brains behind figurative language—A quantitative meta-analysis of neuroimaging studies on metaphor, idiom, and irony processing. Neuropsychologia.

[126] Virshup E., Virshup B. (1980). Visual imagery: The language of the right brain. Imagery.

[127] He K. (2017). A Theory of Creative Thinking.

[128] Rapp A.M., Mutschler D.E., Erb M. (2012). Where in the brain is nonliteral language? A coordinate-based meta-analysis of functional magnetic resonance imaging studies. NeuroImage.

[129] Just M.A., Carpenter P.A., Keller T.A., Eddy W.F., Thulborn K.R. (1996). Brain activation modulated by sentence comprehension. Science.

[130] Prat C.S., Long D.L., Baynes K. (2007). The representation of discourse in the two hemispheres: An individual differences investigation. Brain Lang..

[131] Prat C.S., Just M.A. (2011). Exploring the neural dynamics underpinning individual differences in sentence comprehension. Cereb. Cortex.

[132] Segal D., Gollan T.H. (2018). What’s left for balanced bilinguals? Language proficiency and item familiarity affect left-hemisphere specialization in metaphor processing. Neuropsychology.

[133] Dodds C.M., Morein-Zamir S., Robbins T.W. (2011). Dissociating inhibition, attention, and response control in the frontoparietal network using functional magnetic resonance imaging. Cereb. Cortex.

[134] Haldane M., Cunningham G., Androutsos C., Frangou S. (2008). Structural brain correlates of response inhibition in Bipolar Disorder I. J. Psychopharmacol..

[135] Tomasi D., Ernst T., Caparelli E.C., Chang L. (2006). Common deactivation patterns during working memory and visual attention tasks: An intra-subject fMRI study at 4 Tesla. Hum. Brain Mapp..

[136] Lagopoulos J., Ivanovski B., Malhi G.S. (2007). An event-related functional MRI study of working memory in euthymic bipolar disorder. J. Psychiatry Neurosci..

[137] Miyake A., Just M.A., Carpenter P.A. (1994). Working memory constraints on the resolution of lexical ambiguity: Maintaining multiple interpretations in neutral contexts. J. Mem. Lang..

[138] Pearlmutter N.J., MacDonald M.C. (1995). Individual differences and probabilistic constraints in syntactic ambiguity resolution. J. Mem. Lang..

[139] Farmer T.A., Fine A.B., Misyak J.B., Christiansen M.H. (2017). Reading Span Task Performance, Linguistic Experience, and the Processing of Unexpected Syntactic Events. Q. J. Exp. Psychol..

[140] Bellebaum C., Daum I. (2007). Cerebellar involvement in executive control. Cerebellum.

[141] Desmond J.E., Fiez J.A. (1998). Neuroimaging studies of the cerebellum: Language, learning and memory. Trends Cogn. Sci..

[142] Hayter A., Langdon D., Ramnani N. (2007). Cerebellar contributions to working memory. NeuroImage.

[143] Osaka M., Osaka N., Kondo H., Morishita M., Fukuyama H., Aso T., Shibasaki H. (2003). The neural basis of individual differences in working memory capacity: An fMRI study. NeuroImage.

[144] Baier B., Karnath H.-O., Dieterich M., Birklein F., Heinze C., Müller N.G. (2010). Keeping Memory Clear and Stable—The Contribution of Human Basal Ganglia and Prefrontal Cortex to Working Memory. J. Neurosci..

[145] Ell S.W., Marchant N.L., Ivry R.B. (2006). Focal putamen lesions impair learning in rule-based, but not information-integration categorization tasks. Neuropsychologia.

[146] Cardillo E.R., Watson C., Schmidt G.L., Kranjec A., Chatterjee A. (2012). From novel to familiar: Tuning the brain for metaphors. NeuroImage.

[147] Subramaniam K., Faust M., Beeman M., Mashal N. (2012). The Repetition Paradigm: Enhancement of novel metaphors and suppression of conventional metaphors in the left inferior parietal lobe. Neuropsychologia.

[148] Leung H.C., Gore J.C., Goldman-Rakic P.S. (2002). Sustained mnemonic response in the human middle frontal gyrus during on-line storage of spatial memoranda. J. Cogn. Neurosci..

[149] Abutalebi J., Annoni J.-M., Zimine I., Pegna A., Seghier M., Lee-Jahnke H., Lazeyras F., Cappa S., Khateb A. (2007). Language Control and Lexical Competition in Bilinguals: An Event-Related fMRI Study. Cereb. Cortex.

[150] Abutalebi J., Green D. (2007). Bilingual language production: The neurocognition of language representation and control. J. Neurolinguist..

[151] Lakoff G., Johnson M. (1980). The metaphorical structure of the human conceptual system. Cogn. Sci..

[152] Gibbs R.W., Bogdanovich J.M., Sykes J.R., Barr D.J. (1997). Metaphor in Idiom Comprehension. J. Mem. Lang..

[153] Chen Y., Lai H. (2012). EFL learners’ awareness of metonymy-metaphor continuum. Lang. Aware..

[154] Ruigrok A.N., Salimi-Khorshidi G., Lai M.-C., Baron-Cohen S., Lombardo M., Tait R.J., Suckling J. (2013). A meta-analysis of sex differences in human brain structure. Neurosci. Biobehav. Rev..

[155] Shaywitz B.A., Shaywltz S.E., Pugh K.R., Constable R.T., Skudlarski P., Fulbright R.K., Bronen R.A., Fletcher J.M., Shankweiler D.P., Katz L. (1995). Sex differences in the functional organization of the brain for language. Nature.

[156] Horga G., Kaur T., Peterson B.S. (2014). Annual Research Review: Current limitations and future directions in MRI studies of child-and adult-onset developmental psychopathologies. J. Child Psychol. Psychiatry.

[157] Juffs A. (2004). Representation, Processing and Working Memory in a Second Language. Trans. Philol. Soc..

[158] Juffs A. (2006). Working memory, second language acquisition and low-educated second language and literacy learners. LOT Occas. Ser..

[159] Juffs A., Harrington M. (2011). Aspects of working memory in L2 learning. Lang. Teach..

[160] Sagarra N. (2007). From CALL to face-to-face interaction: The effect of computer-delivered recasts and working memory on L2 development. Conversational Interaction in Second Language Acquisition: A Collection of Empirical Studies.

[161] Havik E., Roberts L., Van Hout R., Schreuder R., Haverkort M. (2009). Processing Subject-Object Ambiguities in the L2: A Self-Paced Reading Study with German L2 Learners of Dutch. Lang. Learn..

[162] Badre D., Wagner A.D. (2002). Semantic retrieval, mnemonic control, and prefrontal cortex. Behav. Cogn. Neurosci. Rev..

[163] Gabrieli J.D.E., Poldrack R.A., Desmond J.E. (1998). The role of left prefrontal cortex in language and memory. Proc. Natl. Acad. Sci. USA.

